# Identification and validation of SHC1 and FGFR1 as novel immune-related oxidative stress biomarkers of non-obstructive azoospermia

**DOI:** 10.3389/fendo.2024.1356959

**Published:** 2024-09-26

**Authors:** Yang Pan, Xiangyu Chen, Hang Zhou, Mingming Xu, Yuezheng Li, Qihua Wang, Zhunan Xu, Congzhe Ren, Li Liu, Xiaoqiang Liu

**Affiliations:** Department of Urology, Tianjin Medical University General Hospital, Tianjin, China

**Keywords:** non-obstructive azoospermia, immune cell, oxidative stress, WGCNA, machine learning, single-cell RNA-seq

## Abstract

**Background:**

Non-obstructive azoospermia (NOA) is a major contributor of male infertility. Herein, we used existing datasets to identify novel biomarkers for the diagnosis and prognosis of NOA, which could have great significance in the field of male infertility.

**Methods:**

NOA datasets were obtained from the Gene Expression Omnibus (GEO) database. CIBERSORT was utilized to analyze the distributions of 22 immune cell populations. Hub genes were identified by applying weighted gene co-expression network analysis (WGCNA), machine learning methods, and protein–protein interaction (PPI) network analysis. The expression of hub genes was verified in external datasets and was assessed by receiver operating characteristic (ROC) curve analysis. Gene set enrichment analysis (GSEA) was applied to explore the important functions and pathways of hub genes. The mRNA–microRNA (miRNA)–transcription factors (TFs) regulatory network and potential drugs were predicted based on hub genes. Single-cell RNA sequencing data from the testes of patients with NOA were applied for analyzing the distribution of hub genes in single-cell clusters. Furthermore, testis tissue samples were obtained from patients with NOA and obstructive azoospermia (OA) who underwent testicular biopsy. RT-PCR and Western blot were used to validate hub gene expression.

**Results:**

Two immune-related oxidative stress hub genes (*SHC1* and *FGFR1*) were identified. Both hub genes were highly expressed in NOA samples compared to control samples. ROC curve analysis showed a remarkable prediction ability (AUCs > 0.8). GSEA revealed that hub genes were predominantly enriched in toll-like receptor and Wnt signaling pathways. A total of 24 TFs, 82 miRNAs, and 111 potential drugs were predicted based on two hub genes. Single-cell RNA sequencing data in NOA patients indicated that *SHC1* and *FGFR1* were highly expressed in endothelial cells and Leydig cells, respectively. RT-PCR and Western blot results showed that mRNA and protein levels of both hub genes were significantly upregulated in NOA testis tissue samples, which agree with the findings from analysis of the microarray data.

**Conclusion:**

It appears that *SHC1* and *FGFR1* could be significant immune-related oxidative stress biomarkers for detecting and managing patients with NOA. Our findings provide a novel viewpoint for illustrating potential pathogenesis in men suffering from infertility.

## Introduction

Infertility is defined as unsuccessful pregnancy after engaging in normal, unprotected sexual intercourse for more than 1 year (1). Approximately 30% of couples worldwide are infertile, and almost half of the cases are attributed to male factors (2). There is significant evidence to indicate that the incidence of male infertility has increased in recent years. Azoospermia is diagnosed as the inability to identify a single sperm cell in three consecutive ejaculations (3). As such, azoospermia is defined as an absolute spermatozoa deficiency in the ejaculate, even on microscopic examination of a cell pellet obtained by centrifugation of the semen sample (4). Azoospermia is a key cause of infertility in men; this condition can be defined as either obstructive azoospermia (OA) or non-obstructive azoospermia (NOA). NOA is found in approximately 10% of infertile men and 1% of the general male population (5), making it a primary cause of male infertility (6). The pathogenesis of NOA includes genetic, developmental, hormonal, environmental, and other reasons, and its management remains challenging (7). Following the development of testicular sperm extraction (TESE), the combination of TESE and intracytoplasmic sperm injection (ICSI) has been regarded as a primary management strategy for male patients with NOA (5, 8). However, retrieval of spermatozoa by TESE is only successful in approximately 50% of NOA cases, due largely to the heterogeneity of NOA. As a result, exploration of the exact pathogenesis and molecular markers for managing NOA cases is important.

The rapid advancement of high-throughput sequence techniques has greatly facilitated research on the genetic characteristics of spermatogenesis. EAU guidelines also illustrate the importance of karyotype abnormalities and Y chromosome microdeletions in NOA cases (4). While numerous reports have shown that a variety of genetic mutations could be associated with NOA, the failure of spermatogenesis may also be linked to various unknown factors (9). Overall, the pathogenesis of NOA is relatively complex and may result from the alternation of multiple genes rather than a single monogenetic factor. Identifying novel biomarkers with high statistical efficiency could hold significant value for assessing the clinical outcomes of patients with NOA.

Many previous studies have revealed that NOA was associated with immune (10, 11) and oxidative stress in testis tissues and cells (12, 13). Gene microarray assessment of testis tissues could aid in identifying novel biomarkers for diagnosing and predicting prognosis in patients with NOA. In the present study, a weighted gene co-expression network analysis (WGCNA) method was applied to accurately explore the complicated molecular mechanisms and identify potential immune- and oxidative stress-associated gene biomarkers for NOA. WGCNA is commonly utilized to identify highly correlated module genes and confirm hub genes associated with phenotype characteristics (14). This novel method has been widely applied by a diverse range of biological researchers in the field of molecular genetics, cancer, and others. WGCNA differs from traditional analyses of microarray-generated differentially expressed genes (DEGs). This novel method can create various co-expression clusters to contextualize high-dimensional microarray data into fewer variables, presenting an enhanced view of associating gene clusters with phenotypes.

In the present study, we combined WGCNA and three machine learning methods to identify novel immune- and oxidative stress-related biomarkers for NOA based on testis tissue expression microarray. Subsequently, single-cell RNA sequencing data from the testes of patients with NOA were used to validate hub gene distribution in single-cell clusters. Furthermore, testis tissue samples were obtained from patients who underwent testicular biopsy. Hub gene expression levels were further validated by real-time polymerase chain reaction (RT-PCR) and Western blot. Overall, this study provides insight into the underlying pathogenesis and can contribute to the identification of crucial medicinal targets for patients with NOA suffering from infertility.

## Materials and methods

### Datasets

**Figure 1:**
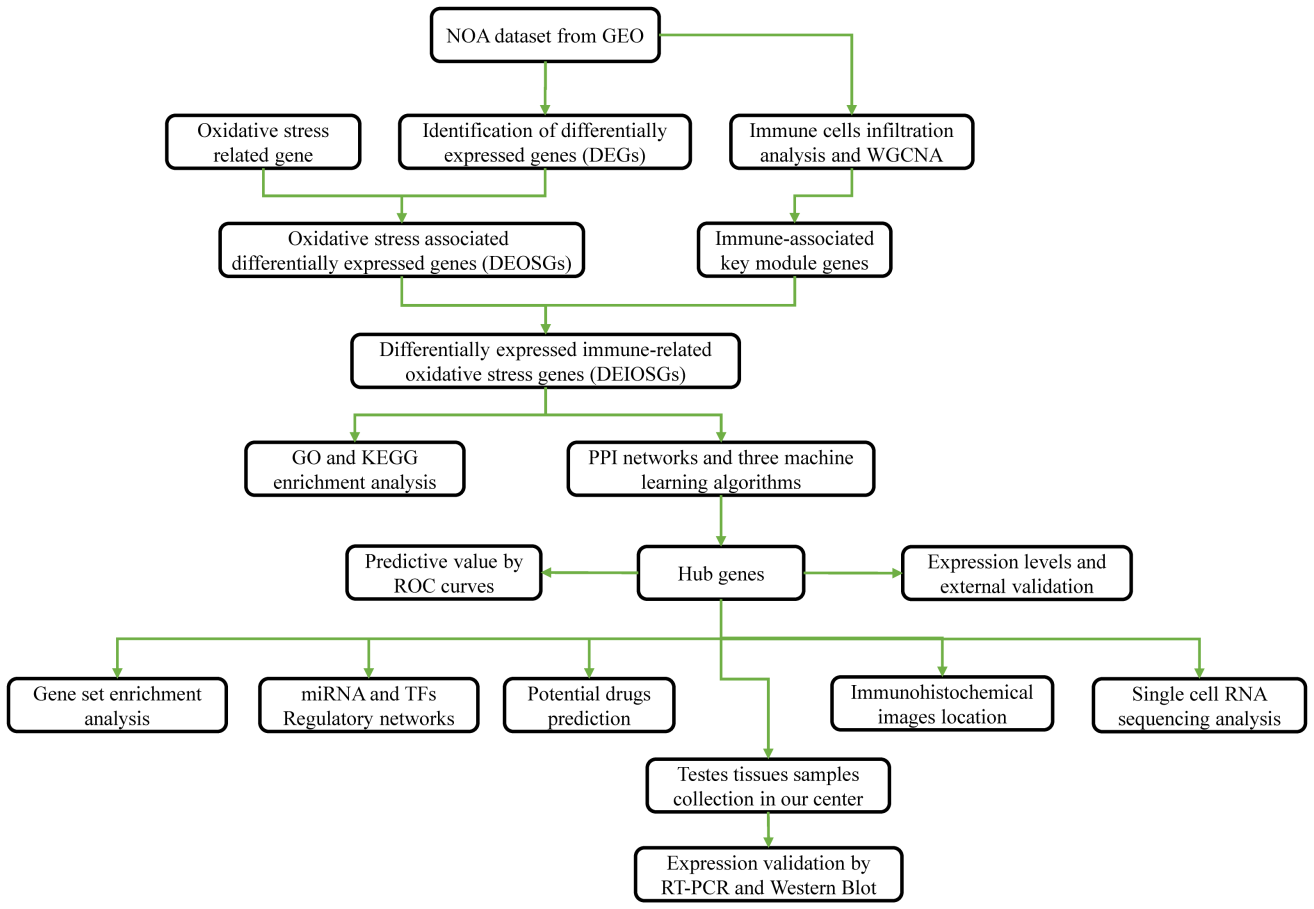
Flowchart for this study.

**Figure 1** presents a flowchart of this study. NOA microarray data were obtained from the Gene Expression Omnibus (GEO) database (http://www.ncbi.nlm.nih.gov/geo/). We selected the dataset GSE9210 to further identify hub genes, as it contained the largest sample size, including data from 47 patients with NOA and 11 OA controls.

### Identification of differentially expressed genes

The “limma” package (15) with the standard of |log_2_ (Fold Change) | > 1 and *p-*value < 0.05 was utilized to identify DEGs. The “pheatmap” and “ggplot2” packages were utilized to plot heatmap and volcanic map of DEGs, separately.

Moreover, 1,792 genes related to oxidative stress were obtained from the GeneCards database (https://www.genecards.org/) with a criterion of >5 scores. The 1,792 genes were intersected with DEGs to generate the differentially expressed oxidative stress-associated genes (DEOSGs).

### Immune infiltration analysis and WGCNA constructio

CIBERSORT uses a deconvolution algorithm to assess the composition and abundance of immune cells according to microarray data (16). An online website, CIBERSORTx (https://cibersortx.stanford.edu/), was utilized to determine the distribution matrix of different immune cell populations between NOAs and controls in the GSE9210 dataset. The WGCNA method was used to identify candidate disease biomarkers, as described in our previous study (17). The “WGCNA” package was further used to perform WGCNA and identify the modules most strongly related to immune cells in patients with NOA. The sample data were preprocessed and outliers were removed. Subsequently, the “WGCNA” package was applied to create the correlation matrix. The optimal soft threshold was selected to convert the correlation matrix into an adjacency matrix, and a topological overlap matrix (TOM) was created from the adjacency matrix. The TOM-based phase dissimilarity metric was utilized to categorize genes with similar expression patterns into gene modules using average linkage hierarchical clustering. The module with the significantly highest correlation to immune cells was regarded as the core module. Eventually, the genes in the core module were intersected with DEOSGs. The intersected genes were identified as differentially expressed immune-related oxidative stress genes (DEIOSGs) for subsequent analysis.

### GO and KEGG enrichment analysis

To evaluate the functional and pathway outcomes of DEIOSGs, GO and KEGG enrichment were analyzed using the “clusterProfiler” package (18, 19). The “ggplot2” package was utilized to visualize enrichment analysis outcomes.

### Identification of hub genes

Three machine learning algorithms—least absolute shrinkage and selection operator (LASSO) regression (20), support vector machine–recursive feature elimination (SVM-RFE) (21), and random forest (RF) (22)—were applied to identify hub genes based on DEIOSGs. The genes from each machine learning method were intersected to identify the common hub genes.

Furthermore, protein–protein interaction (PPI) networks based on DEIOSGs were generated using the STRING database (https://cn.string-db.org/). Subsequently, PPI networks were analyzed using the cytoHubba plugin in the Cytoscape software (v3.7.2). Twelve algorithms in the cytoHubba plugin were performed, after which the top 10 genes from every algorithm were intersected to identify common genes, as another hub gene.

Eventually, the genes identified by these two methods were regarded as hub genes.

### Gene set enrichment analysis

Single-gene gene set enrichment analysis (GSEA) was conducted to explore the potential function of each hub gene using the “clusterProfiler” and “org.Hs.eg.db” packages (23).

### Constructing the mRNA–miRNA–TFs network and predicting drugs

The JASPAR and TarBase databases extracted from the NetworkAnalyst (https://www.networkanalyst.ca/) were used to forecast hub genes encoding transcription factors (TF) and microRNAs (miRNA), respectively. In addition, the DSigDB database through the Enrichr platform (https://amp.pharm.mssm.edu/Enrichr/) was applied to forecast potential drugs.

### Hub gene expression analysis and validation

Hub gene expression comparisons between the NOA and control groups were explored using data extracted from the GSE9210 database. To validate hub gene expression trends, we obtained another dataset (GSE145467), which included 10 NOA samples and 10 OA control samples. The Mann–Whitney test was applied to compare hub gene expressions.

### Predictive value for NOA using hub genes

To evaluate the performance of hub genes, we assessed their ability to predict clinical phenotype by constructing receiver operating characteristic (ROC) curves using the “timeROC” package. Furthermore, samples from the validation set (GSE145467) were utilized to verify predictive accuracy. The predictive value of hub genes in each dataset was assessed using the areas under ROC curves (AUCs).

### Immunohistochemical analysis

Immunohistochemical data and images were obtained from the Human Protein Atlas (HPA) database (https://www.proteinatlas.org/) to explore hub gene distribution in testis tissues.

### Single-cell RNA sequencing analysis

A single-cell RNA sequencing (scRNA-seq) database for testis diseases, named the Male Health Atlas database (http://malehealthatlas.cn/), was utilized to assess hub gene distribution in single-cell clusters of testis tissues from patients with NOA.

### Testis tissue sample collection

Testis tissue samples were obtained from patients with NOA and OA controls who underwent testicular biopsy. Samples were immediately flash-frozen in liquid nitrogen for preservation. All patient procedures were performed in accordance with the Declaration of Helsinki and were approved by the Ethics Committee. Written informed consent was obtained from all cases in our study.

### Expression validation by RT-PCR

Total RNA from testes was extracted using TRIzol reagent (Invitrogen) as per the manufacturer’s instructions. cDNA was synthesized using commercial kits. A qPCR commercial kit was also utilized to amplify transcript and analyze the mRNA levels of genes in testes. The reaction was performed in a 20-μL volume comprising 10 μL of mix, 10 μM forward and reverse primers, 1 μg of diluted cDNA sample, and RNase-free water. The optimal PCR conditions were 95°C for 120 s, followed by 40 cycles of 95°C for 15 s and 60°C for 30 s. After checking for reference gene suitability, beta-actin was utilized as an internal control gene. The relative mRNA expression levels of target genes were normalized to beta-actin, and results were analyzed using the 2^–ΔΔCT^ method. The primers were purchased from Sangon Biotech (Shanghai). All primers were tested and quality controlled by this company and met the standards. The primers are shown in Additional file 1 (**Supplementary Table S1**).

### Expression validation by Western blot

Total protein was extracted as per the manufacturer’s instructions (cat. no. R0020; Solarbio, China). Protein concentration was measured using BCA Protein Quantitation Assay (cat. no. PC0020; Solarbio, China). Protein samples were separated using SDS-PAGE and transferred onto polyvinylidene fluoride (PVDF) membranes (cat. no. FFP26, Beyotime, China). The membranes were then blocked in non-fat powdered milk (cat. no. D8340; Solarbio, China) and incubated overnight in primary antibodies at 4°C. Primary antibodies included rabbit anti-beta-actin (1:5,000; cat. no. bs-0061R; Bioss, China), rabbit anti-FGFR1 (1:1,000; cat no. 60325-1-Ig; Proteintech, USA), and rabbit anti-SHC1 (1:1,000; cat. no. NBP3-21850; Novus Biologicals, USA). Subsequently, the PVDF membranes were incubated in the secondary antibody (1:5,000; cat. no. bs-0295G-HRP; Bioss, China) for 1 h, then washed three times in TBS-T. Finally, the ECL Plus kit (cat. no. PE0010; Solarbio, China) was applied to expose the immunoreactive blots. ImageJ software was used to scan the blot density. Hub gene protein expression was normalized to the expression of beta-actin.

### Statistical analysis

R Project software (version 4.2.2) was used to analyze and visualize the data. Graphics were created using GraphPad Prism 8.0 software. *p*-values < 0.05 were considered as statistically significant.

## Results

### Oxidative stress-associated differentially expressed genes

**Figure 2:**
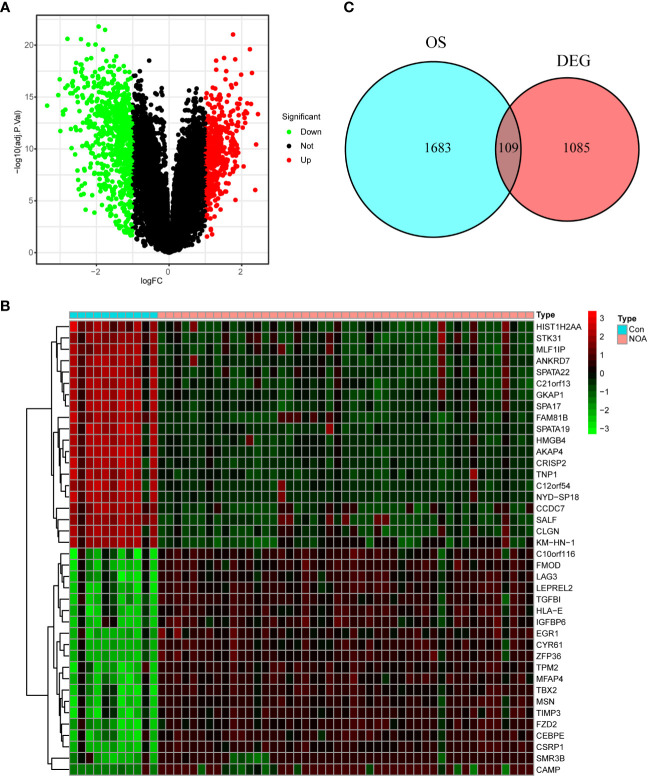
Identification of oxidative stress-associated differentially expressed genes (DEOSGs). **(A)** Volcano plot of differentially expressed genes (DEGs). **(B)** Heatmap of the top 40 DEGs. **(C)** Venn diagrams of generating DEOSGs.

In total, 1,194 DEGs were identified from the GSE9210 dataset. The volcanic map of all DEGs (**Figure 2A**) and the heatmap of the top 40 DEGs (**Figure 2B**) were constructed. Moreover, 1,792 oxidative stress-associated genes were obtained from the GeneCards database with a criterion of >5 scores. After the intersection, 109 DEOSGs were identified (**Figure 2C**).

### Immune cell infiltration analysis and WGCNA results

**Figure 3:**
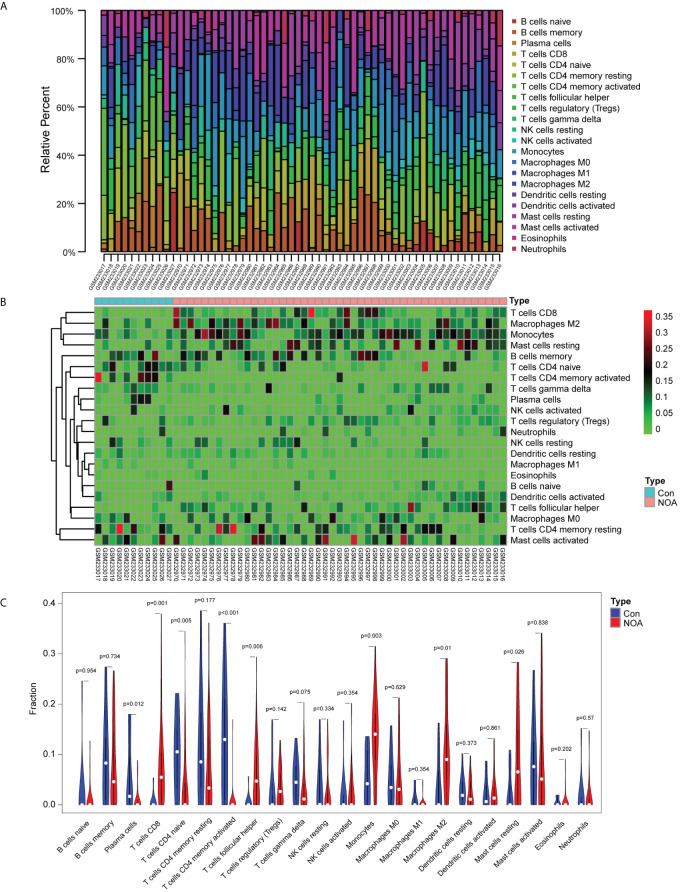
Immune infiltration analysis based on the CIBERSORT algorithm and non-obstructive azoospermia (NOA) dataset. **(A)** Relative percentage of 22 immune cells in each sample. **(B)** Heatmap of 22 immune cells in each sample. **(C)** Comparison of 22 immune cells between non-obstructive azoospermia (NOA) samples and control samples.

Application of the CIBERSORT algorithm revealed that 8/22 immune cells (plasma cells, CD8^+^ T cells, CD4^+^ naïve T cells, CD4^+^ memory-activated T cells, follicular helper T cells, monocytes, M2 macrophages, and resting mast cells) were found to be significantly different between the NOA and OA control groups (**Figures 3A–C**).

**Figure 4:**
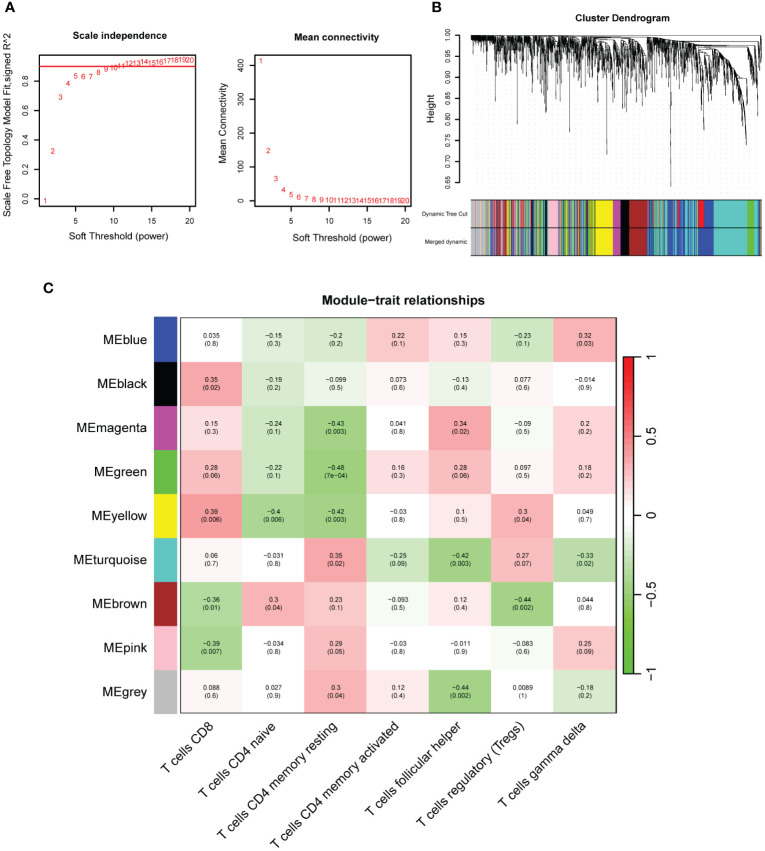
Construction of weighted gene co-expression networks analysis (WGCNA). **(A)** Choosing the best soft-threshold power. **(B)** Dynamic tree cut and merged dynamic in WGCNA. **(C)** Nine immune-related gene modules revealed by the WGCNA.

**Figure 5:**
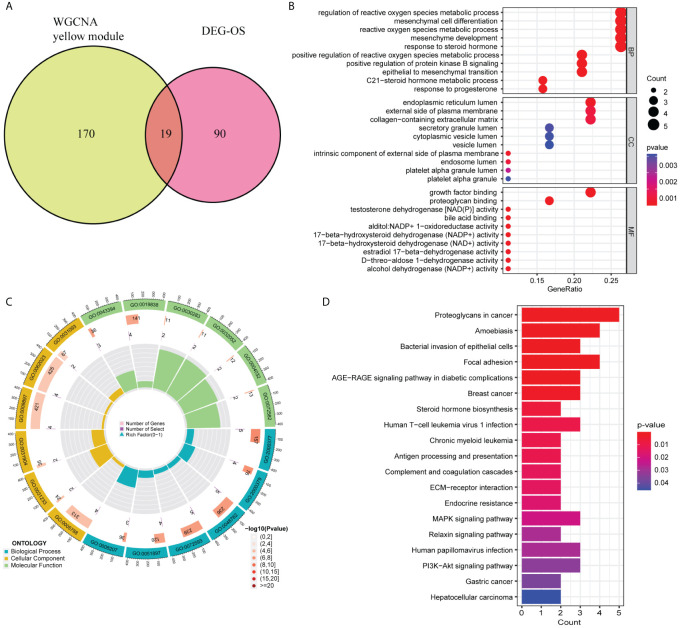
Gene Ontology (GO) and Kyoto Encyclopedia of Genes and Genomes (KEGG) enrichment analysis based on 19 differentially expressed immune-related oxidative stress genes (DEIOSGs). **(A)** Venn diagrams of generating DEIOSGs. **(B)** Bubble plot of GO enrichment analysis outcomes. **(C)** Circle plot of GO enrichment analysis outcomes. **(D)** Bar plot of KEGG enrichment analysis outcomes.

WGCNA revealed that the soft-threshold power was calibrated to 9 (*R*^2^ = 0.86) (**Figure 4A**), and a total of nine modules were identified (**Figures 4B, C**). Among them, the yellow module had the most strongly positive correlations with CD8^+^ T cells (**Figure 4C**). Owing to its significance in relation to immune infiltrating cells, the yellow module including 189 genes was chosen for further investigation. Nineteen DEIOSGs were identified as the intersection between the 109 DEOSGs and the yellow module genes (**Figure 5A**).

### GO and KEGG

GO and KEGG enrichment analysis based on 19 DEIOSGs were conducted. The biological processes of GO analysis were engaged in the regulation of reactive oxygen species metabolic process, mesenchymal cell differentiation, and other functions. The cellular components of the GO analysis included endoplasmic reticulum lumen, the external side of the plasma membrane, and collagen-containing extracellular matrix. The molecular functions of GO analysis predominantly included growth factor binding, proteoglycan binding, and testosterone dehydrogenase activity (**Figures 5B, C**). KEGG analysis of 19 DEIOSGs was predominantly engaged in human papillomavirus infection, MAPK signaling pathway, steroid hormone biosynthesis, and other pathways (**Figure 5D**).

### Identification of hub genes

**Figure 6:**
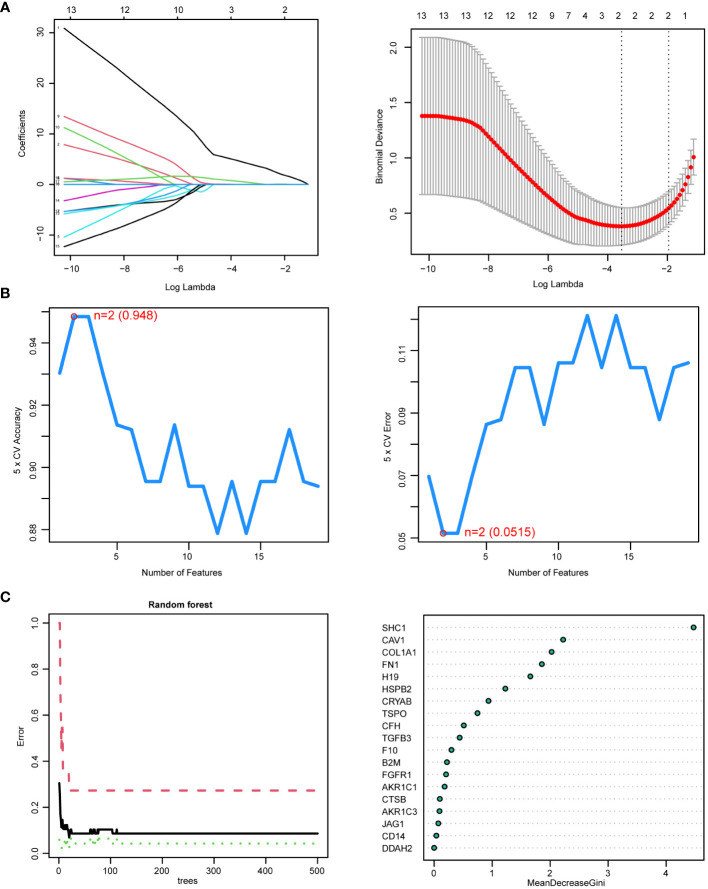
Hub genes identified by three machine learning algorithms based on 19 differentially expressed immune-related oxidative stress genes (DEIOSGs). **(A)** Outcomes of least absolute shrinkage and selection operator (LASSO) regression algorithm. (Left) LASSO plot showed that the variations in the size of coefficients for parameters shrank as the value of k penalty increased. (Right) LASSO logic coefficient penalty diagram. **(B)** Outcomes of the support vector machine–recursive feature elimination (SVM-RFE) algorithm. (Left) The relationship between the prediction accuracy of SVM-RFE and the number of features. (Right) The relationship between the prediction error rate of SVM-RFE and the number of features. **(C)** Outcomes of random forest (RF) algorithm. (Left) The error rate confidence intervals for random forest mode. (Right) The dot graph illustrating the relative importance of genes in the random forest model.

**Figure 7:**
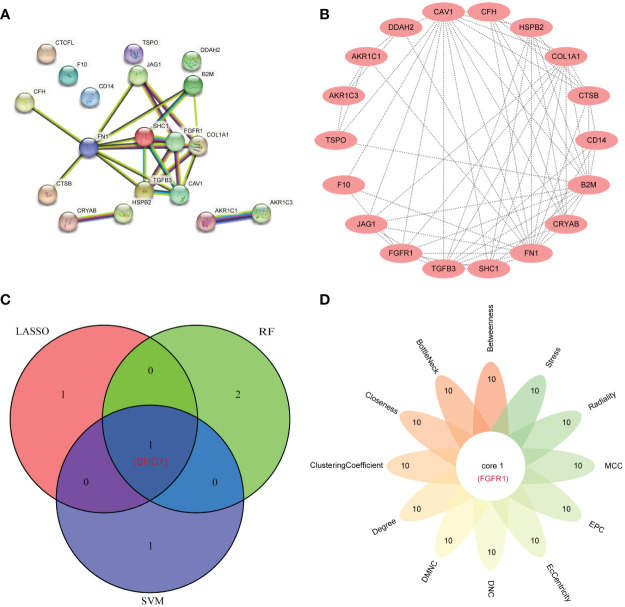
Hub gene identification by the protein–protein interaction (PPI) network. **(A)** The PPI network from the STRING database based on 19 differentially expressed immune-related oxidative stress genes (DEIOSGs). **(B)** PPI network visualization using the Cytoscape software. **(C)** Venn diagrams for intersecting the genes from three machine learning methods. **(D)** Flower Venn diagrams for intersecting the genes from 12 algorithms using the cytoHubba plugin in the Cytoscape software.

From the 19 DEIOSGs, 2, 2, and 3 genes were identified using LASSO (**Figure 6A**), SVM-RFE (**Figure 6B**), and RF (**Figure 6C**) analyses, respectively. Intersection of these gene lists identified one common gene, *SHC1* (**Figure 7C**). Furthermore, we identified another hub gene, *FGFR1*, through the intersection of genes from 12 cytoHubba algorithms in PPI networks (**Figures 7A, B, D**). Eventually, these two hub genes (*SHC1* and *FGFR1*) were identified as novel immune-related oxidative stress biomarkers for NOA.

### Expression validation

**Figure 8:**
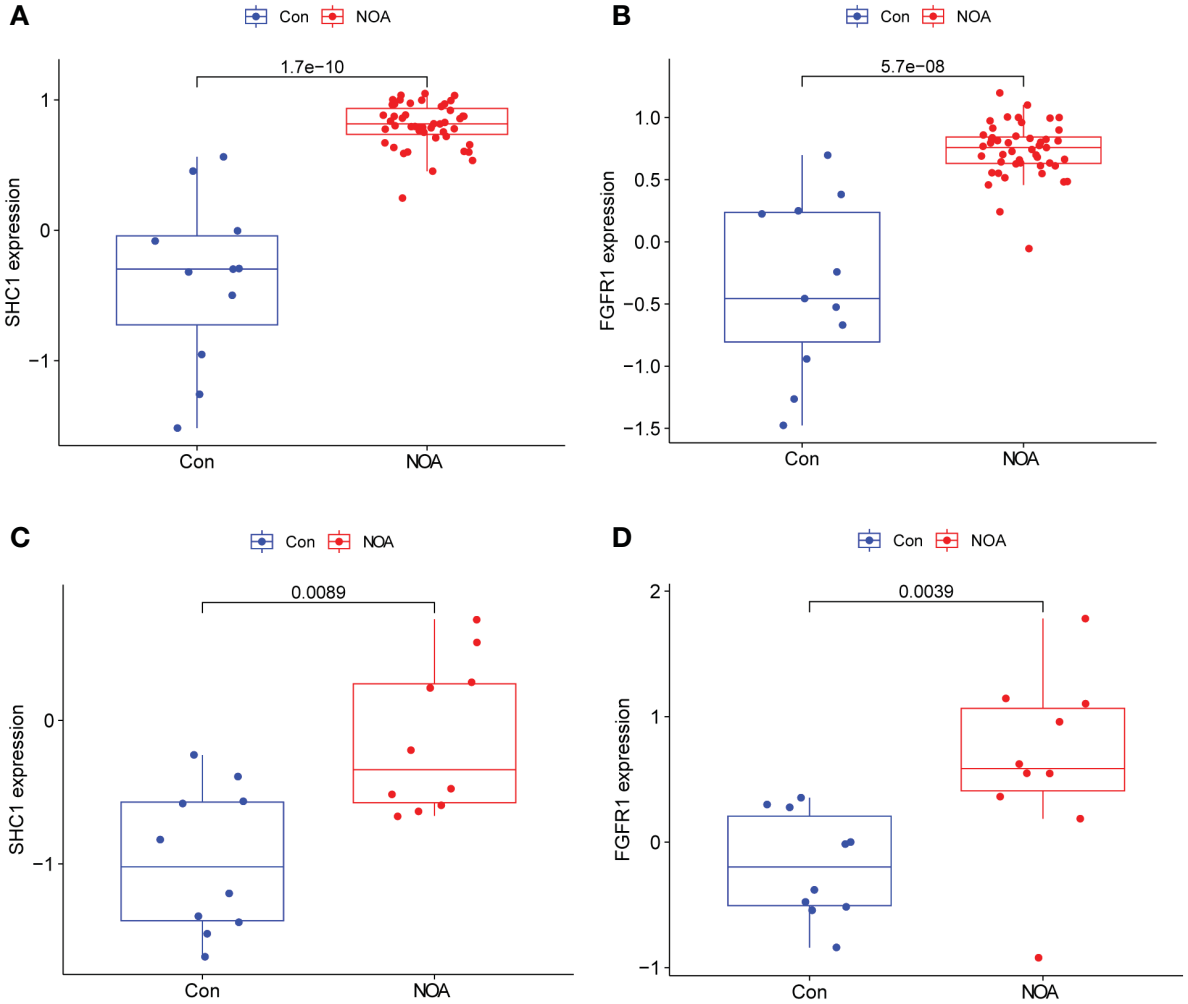
Hub gene expression and external validation. **(A, B)** Expression of two hub genes in the GSE9210 dataset. **(C, D)** Expression validation of two hub genes in an external dataset, GSE145467.

In NOA samples, the expression of the two hub genes was found to be significantly higher than those in the OA control samples (**Figures 8A, B**). This observation was further confirmed by analyzing an external NOA dataset (GSE145467), which further revealed that two hub genes were more highly expressed in NOA samples compared to OA control samples (**Figures 8C, D**). All of these comparisons were statistically significant.

### ROC curve for predicting the NOA phenotype

**Figure 9:**
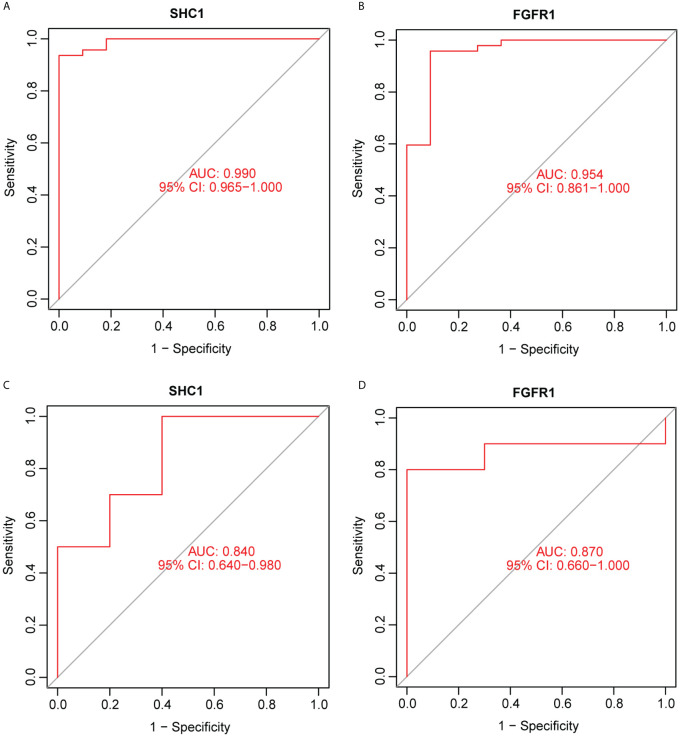
Receiver operating characteristic (ROC) curve for predicting the non-obstructive azoospermia (NOA) phenotype using hub genes. **(A, B)** Two hub genes were analyzed using ROC curves for predicting the NOA phenotype in the GSE9210 dataset. **(C, D)** Two hub genes were analyzed using ROC curves for predicting the NOA phenotype based on an external dataset, GSE145467.

ROC curve analysis was performed and the AUCs were calculated to detect the diagnostic accuracy of the two hub genes. To predict the NOA phenotype, AUCs were 0.99 (95% CI: 0.965–1.0) for *SHC1* and 0.954 (95% CI: 0.861–1.0) for *FGFR1* (**Figures 9A, B**) in the integrated training group (GSE9210). This observation was further confirmed through analysis of an external NOA dataset (GSE145467), which revealed that the AUCs of *SHC1* and *FGFR1* were 0.84 (95% CI: 0.64–0.98) and 0.87 (95% CI: 0.66–1.000), respectively (**Figures 9C, D**). All AUCs in these datasets for predicting the NOA phenotype were relatively high.

### Single-gene GSEA assessment

**Figure 10:**
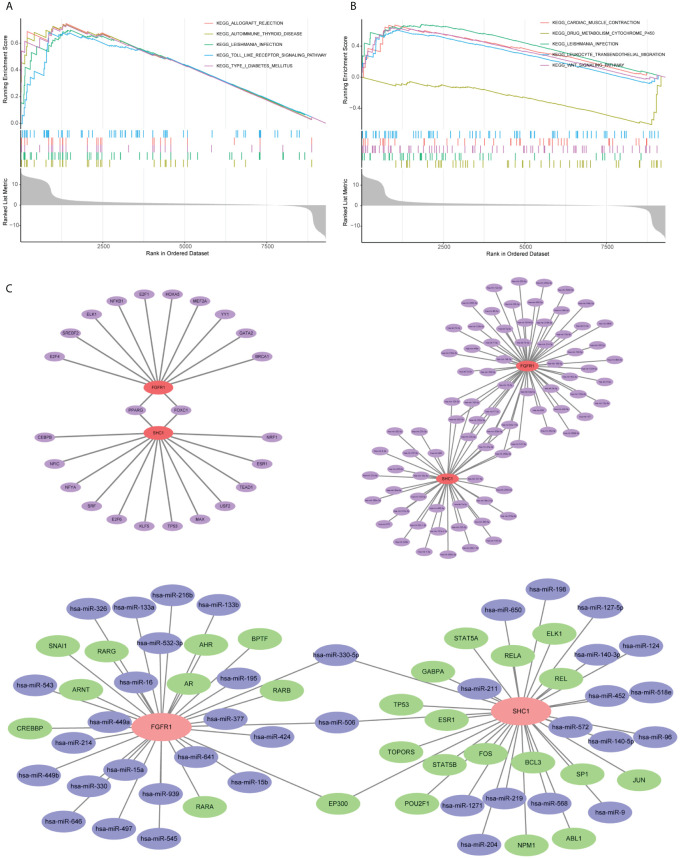
Single-gene gene set enrichment analysis (GSEA) and mRNA–microRNA–transcription factors (TFs) regulatory network construction based on two hub genes. **(A)** GSEA outcome of SHC1. **(B)** GSEA outcome of FGFR1. **(C)** mRNA–microRNA–TFs regulatory network construction.

GSEA of the hub gene SHC1 was predominantly enriched in allograft rejection, autoimmune thyroid disease, leishmania infection, toll-like receptor (TLR) signaling pathway, and type I diabetes mellitus (**Figure 10A**). GSEA of the hub gene *FGFR1* revealed predominant enrichment in cardiac muscle contraction, drug metabolism cytochrome P450, leishmania infection, leukocyte trans-endothelial migration, and the WNT signaling pathway (**Figure 10B**).

### Regulatory network construction and potential drug prediction

Evaluation of the two hub genes in the JASPAR database identified 24 TFs (**Figure 10C**). Among them, two (*FOXC1* and *PPARG*) had a degree of ≥2. Using the TarBase database, 82 miRNAs were predicted (**Figure 10C**). Among them, 11, including has-mir-200b-3p and has-mir-124-3p, had a degree of ≥2. An overall regulatory network comprising hub genes, TFs, and miRNAs was further constructed (**Figure 10C**). Moreover, a total of 111 potential drugs were predicted from the DSigDB database based on the two hub genes (Additional file 1 **Supplementary Table S2**).

### Immunohistochemical location

**Figure 11:**
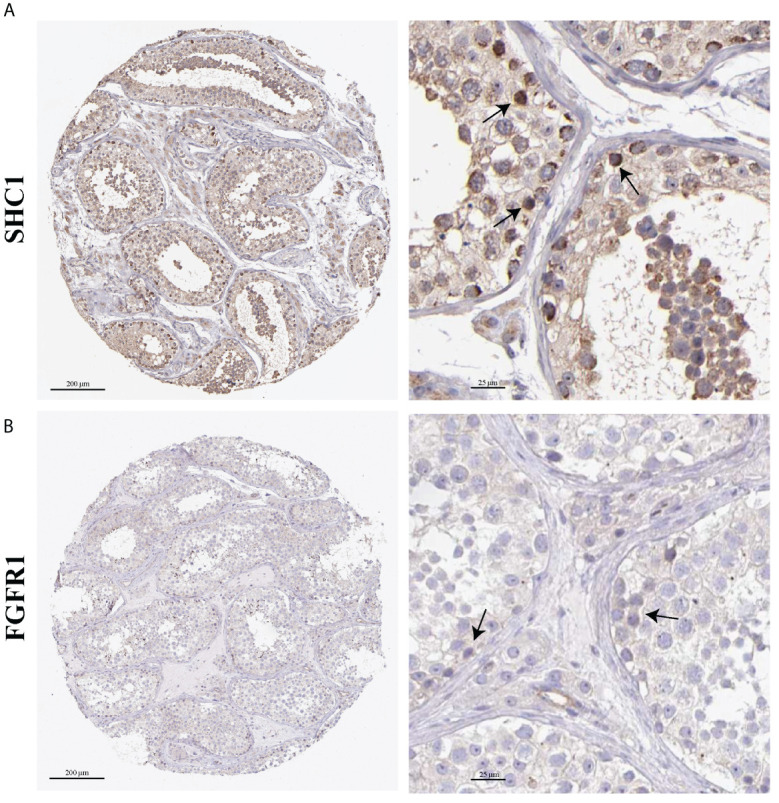
Immunohistochemistry images on hub gene expression based on testis tissues from the Human Protein Atlas (HPA) database. **(A)** Representative immunohistochemistry results of SHC1. **(B)** Representative immunohistochemistry results of FGFR1. Black arrow indicates positive cells.

The localization distribution of hub genes in human testis tissues was explored in the HPA database. *SHC1* was highly positive in spermatogonia (**Figure 11A**). *SHC1* was moderately expressed in Leydig cells, Sertoli cells, pachytene spermatocytes, preleptotene spermatocytes, and round or early spermatids. However, *SHC1* expression was negative in peritubular cells and elongated or late spermatids. Moreover, *FGFR1* was lowly expressed in the seminiferous tubules and Leydig cells (**Figure 11B**).

### scRNA-seq data analysis

**Figure 12:**
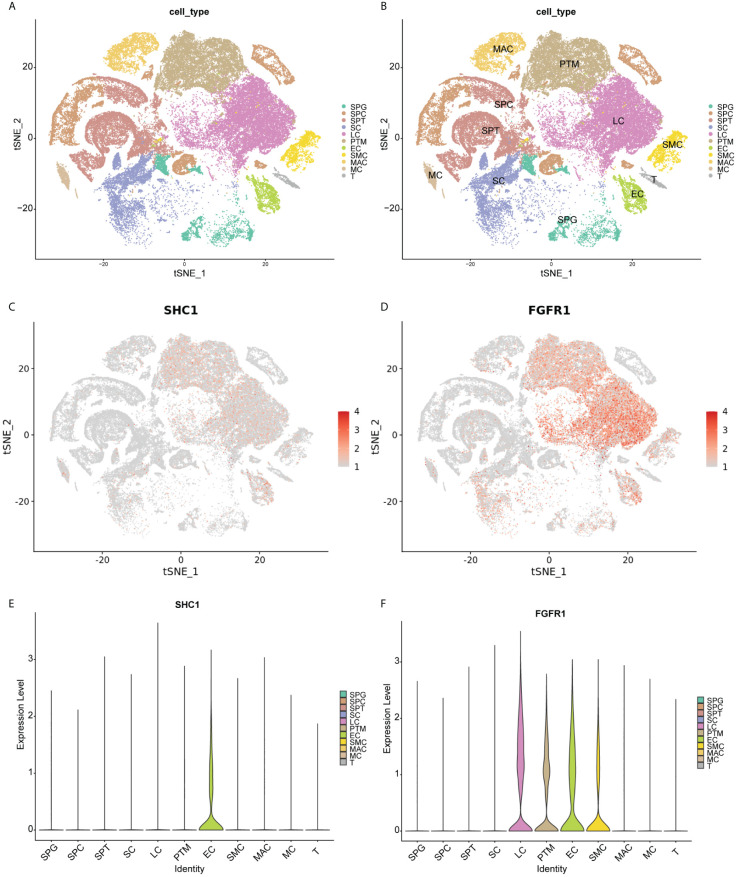
Single-cell RNA sequencing data analysis. **(A, B)** T-distribution stochastic neighbor embedding (TSNE) analysis outcomes of 11 cell clusters based on human testis non-obstructive azoospermia atlas in the Male Health Atlas database. **(C, D)** The expression distribution of two hub genes (SHC1 and FGFR1) in 11 cell clusters. **(E, F)** The violin plot showing the expression distribution of two hub genes (SHC1 and FGFR1) in 11 cell clusters. SPG, spermatogonia; SPC, spermatocyte; SPT, spermatids/sperm; SC, Sertoli cell; LC, Leydig cell; PTM, peritubular myoid cell; EC, endothelial cell; SMC, vascular smooth muscle cell; MAC, macrophage; MC, mast cell; T, T cell.

The scRNA-seq data were analyzed to assess the distribution of *SHC1* and *FGFR1* in 11 cell clusters from the human testis NOA atlas in the Male Health Atlas database (**Figures 12A, B**). The results revealed that *SHC1* was the most highly expressed in endothelial cells (**Figures 12C, E**), while *FGFR1* was the most highly expressed in Leydig, peritubular myoid, endothelial, and vascular smooth muscle cells (**Figures 12D, F**).

### RT-PCR and Western blot validation in clinical samples

**Figure 13:**
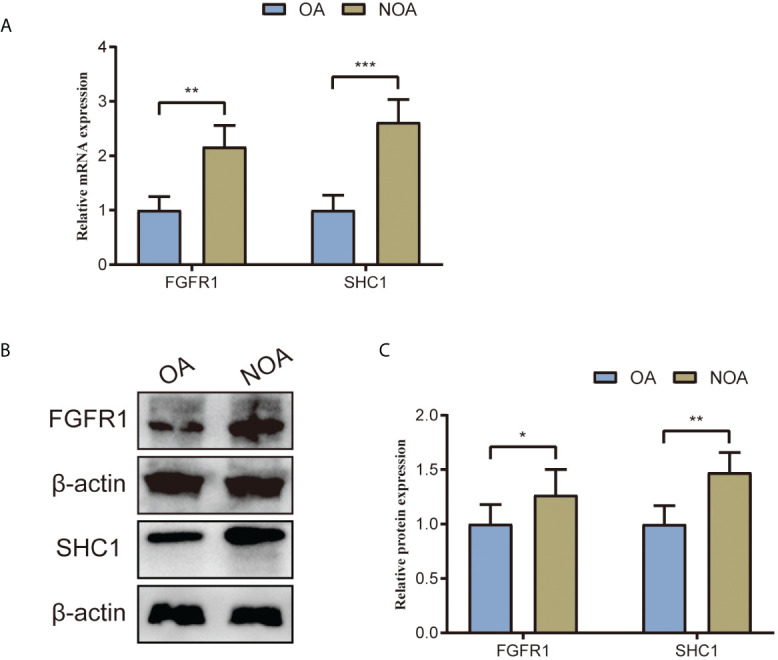
Two hub genes’ (SHC1 and FGFR1) mRNA and protein expression levels validated by RT-PCR and Western blot using testes tissue samples collected from patients with NOA and OA in our center. **(A)** Relative mRNA expression of SHC1 and FGFR1. **(B)** Western blot outcomes of two hub genes (SHC1 and FGFR1). **(C)** Relative protein expression of SHC1 and FGFR1. *p < 0.05; **p < 0.01; ***p < 0.001.

We collected testis tissue samples from patients with NOA and patients with OA who underwent tissue sampling. The gene expression levels of the two hub genes were validated using RT-PCR. RT-PCR results revealed that two hub genes were statistically different between NOA and OA testis tissue samples (**Figure 13A**). Compared to that in OA control samples, mRNA expression of *SHC1* and *FGFR1* were significantly upregulated in NOA testis tissue samples, which is consistent with the findings from the microarray data. Furthermore, we used testis tissue samples of patents with NOA and OA to validate the protein expression levels of the two hub genes using Western blot (**Figure 13B**). Similarly, the protein expression profiles of *SHC1* and *FGFR1* were both significantly upregulated in the NOA group than in the OA control group (**Figure 13C**), which is consistent with the RT-PCR results.

## Discussion

Men with NOA generally present with varying severities and etiologies of abnormalities in spermatogenesis, while the exact pathogenesis remains unclear. Some previous reports have mentioned that genetic alternations could be associated with spermatogenesis abnormalities (24–26). The current management strategies for NOA are limited due to individual differences, necessitating the development of novel molecular markers that can aid in the pathogenesis of NOA. Thus, identifying novel biomarker genes is vital to improve the management of patients with NOA. Immune infiltration and oxidative stress have been found to exert a substantial impact on the progression of NOA (13, 27). In this study, we identified two genes associated with immune and oxidative stress, *SHC1* and *FGFR1*, that serve as novel biomarkers for NOA. ROC curve analysis revealed that both hub genes had outstanding diagnostic value and accuracy. The expression levels and distributions of these hub genes in single-cell clusters were validated through the analysis of an external dataset and the scRNA-seq dataset. Moreover, we collected the testis tissues of patients with NOA and OA controls to validate the mRNA and protein expression by RT-PCR and Western blot. Our findings offer new opportunities for the pathogenesis of NOA and male infertility.

*SHC1* resided within zone 1, region 2 of human chromosome 1 (28). *SHC1* was thought to control the receptor tyrosine kinase pathway and regulate neuronal death (29). Deep investigation into *SHC1* revealed that the gene was crucial for numerous cancer cells (30). Acting as the adapter protein, *SHC1* possessed SH2 domains, which were shared among signal proteins of the cytoplasm (31, 32). This was related to numerous receptor-related signal processes including antigen, hormone, and growth factor (33). In contrast to their non-transformed counterparts, numerous types of transformed cells contained *SHC1*, which was also highly phosphorylated (34). *SHC1* regulates cancer growth owing to its tumor-specific activation, suggesting that it served as a useful predictive marker and a target for therapeutic intervention (35, 36). However, the role of *SHC1* in NOA has not received much attention in the literature. The present study showed that *SHC1* was significantly increased in the testis tissues of NOA cases, indicating an extremely high diagnostic accuracy. Furthermore, functional enrichment analysis indicated that oxidative stress and immune processes were related to *SHC1*, which may be the primary mechanism responsible for *SHC1* participation in NOA.

*FGFR1*, a member of the fibroblast growth factor receptor (FGFR) family, plays a crucial role in biological process (37). Fibroblast growth factor (FGF) binds to the extracellular domain of FGFR, resulting in its activation. Subsequently, receptor dimerization occurs, leading to the phosphorylation of the C-terminal tyrosine (38). The key kinase and pathways were phosphorylated and activated as a result of the activation of the FGF tyrosine kinase family by various FGF ligands, thus regulating multiple physiological responses such as embryogenesis and angiogenesis (39). In various human malignancies, *FGFR1* gene translocation, mutation, and amplification can result in the abnormal activation of the FGFR signaling system, thereby promoting carcinogenesis and tumor progression (40–42). FGF can also promote angiogenesis by activating FGFR1 in endothelial cells (43). For example, FGFR1 activation contributes to the epithelial–mesenchymal transition (EMT) and metastasis in breast cancer (44), as well as the carcinogenesis and EMT of prostate cancer (45). Consequently, FGFR1 was associated with the emergence of many disease and function disorders. However, studies on the involvement of FGFR1 in NOA development are lacking. The present study showed that *FGFR1* was highly expressed in the testes of patients with NOA, and it could be related to the pathogenesis of this condition. This finding opens the door for *FGFR1* to be used as a novel NOA diagnostic marker.

Single-gene GSEA of the hub gene *SHC1* revealed enrichment of the TLR signaling pathway. TLR has the ability to detect the molecular mode associated with causative agents. This detection triggers a series of gene expression alternations, which work in concert to eliminate harmful bacteria (46). The TLR signaling pathway plays a direct role in the activation, growth, differentiation, development, and functioning of T cells in various physiological activities (47). TLR is of utmost importance in the protection against infections, immunodeficiency, and tumor growth. Based on our findings, the TLR signaling pathway may be activated by the high expression of *SHC1* in testis tissues and result in inflammation and activation of the immune response, thus mediating the occurrence of NOA. However, this finding necessitates additional confirmation through *in vivo* and *in vitro* experimentation.

Single-gene GSEA of the hub gene FGFR1 revealed enrichment of the Wnt signaling pathway. The Wnt pathway plays a pivotal role in various evolutionary and illness-related processes. As such, it is a crucial factor in the development and maintenance of organic tissue functions by regulating their native stem cells (48). Recent studies have further shed light on the involvement of the Wnt signaling pathway in the differentiation of human primordial germ cells and the maintenance of mouse spermatogonial stem cells (49–51). Moreover, several previous studies have reported that the downregulation of Wnt signaling in spermatogonia was strongly related to the development of NOA (52). These studies further proposed the significance of the Wnt signaling pathway in inducing human spermatogonial stem cells. Additionally, they indicated that the absence of spermatogonial stem cells in testis tissues of azoospermia could be linked to the inactivation of the Wnt signaling pathway. Similarly, our study also showed that the Wnt signaling pathway significantly decreased in the testis tissues of patients with NOA. Furthermore, our results indicated that FGFR1-medicated Wnt signaling pathway alternations could be a significant aspect of the pathogenesis of NOA.

Our study has some limitations that should be considered. First, our findings are predominantly based on publicly available data. Despite performing expression validation using testicular samples, further experimental validation with larger and multi-center samples is required to validate the results. Second, although the HPA is an extremely helpful tool to verify the protein expression levels in normal tissues, the formalin-fixed material depicts a minor cellular preservation and the spermatogenesis does not seem to be intact. It will be more encouraging to perform immunohistochemistry on the testis samples in the future. Our study revealed that oxidative stress- and immune-related hub genes, *SHC1* and *FGFR1*, could be primary factors in the development of NOA, and these genes are closely interconnected. The findings of immune analysis indicate that mast cells, T cells, monocytes, and macrophages were crucial to the pathogenesis of NOA. Moreover, enrichment analysis indicated that hub genes were primarily concentrated in TLR and Wnt signaling pathways, and were associated with the occurrence of NOA. Thus, focusing on these hub genes and important signaling pathways might be crucial and hopeful. Moreover, in this study, we identified two key genes using machine learning and WGCNA, with validation of their importance performed using single-cell RNA-seq data, RT-PCR, and Western blot. As such, our study has the potential to contribute to a deeper understanding of the pathogenesis of NOA and could further aid in the identification of crucial targets for pharmaceutical exploration.

## Conclusions

This study identified *SHC1* and *FGFR1* as oxidative stress- and immune-related hub genes. These hub genes were significantly upregulated in the testes of patients with NOA and had good predictive values for the NOA phenotype. The TLR and Wnt signaling pathways might be related to the development of NOA. *SHC1* and *FGFR1* could be novel immune-related oxidative stress biomarkers and crucial targets for the pathogenesis of patients with NOA.

## Data availability statement

The datasets presented in this study can be found in online repositories. The names of the repository/repositories and accession number(s) can be found in the article/**Supplementary Material**.

## Ethics statement

The studies involving human participants were reviewed and approved by the Ethics Committee of Tianjin Medical University General Hospital. The patients/participants provided their written informed consent to participate in this study.

## Author contributions

YP: Investigation, Methodology, Writing – original draft. XC: Conceptualization, Validation, Visualization, Writing – review & editing. HZ: Formal Analysis, Visualization, Writing – review & editing. MX: Data curation, Formal Analysis, Software, Writing – review & editing. YL: Visualization, Data curation, Formal Analysis, Writing – review & editing. QW: Validation, Formal Analysis, Writing – review & editing. ZX: Validation, Writing – review & editing. CR: Validation, Visualization, Writing – review & editing. LL: Project administration, Resources, Supervision, Writing – review & editing. XL: Funding acquisition, Project administration, Supervision, Writing – review & editing.
