# Supplementary material for: Identification and validation of SHC1 and FGFR1 as novel immune-related oxidative stress biomarkers of non-obstructive azoospermia
Source: Front Endocrinol (Lausanne). 2024 Sep 26;15:1356959. doi: 10.3389/fendo.2024.1356959 (PMC11466301; doi:10.3389/fendo.2024.1356959)
Supplement: Supplementary file 1 [file DataSheet_1.pdf]

Table S1. Primers information for hub genes.

Genes	Primer	Sequence (5'->3')	Length	Tm
**SHC1**	**Forward**	TGAGGGTGTGGTTCGGACTAAGG	23	63.77
	**Reverse**	CCGCAGA GATGATGGGCAAGTG	22	63.81
**FGFR1**	**Forward**	CTTCGTTTCTTGTTGGTATGC	21	55.81
	**Reverse**	GGACAGGATGGAGTTTGGAC	20	57.88
**β-Actin**	**Forward**	GATTACTGCTCTGGCTCCTAGC	22	60.29
	**Reverse**	GACTCATCGTACTCCTGCTTGC	22	61.05

Table S2. Potential drugs were predicted from DSigDB database based on hub genes.

Term	Overlap	P-value	Adjusted P-value	Old P-value	Old Adjusted P-value	Odds Ratio	Combined Score	Genes
Arsenic acid CTD 00001208	1/11	0.0011	0.019071	0	0	1998.8	13617.26	SHC1
Ro-4396686 TTD 00010666	1/12	0.0012	0.019071	0	0	1817	12220.65	FGFR1
urea CTD 00006965	1/13	0.0013	0.019071	0	0	1665.5	11068.43	FGFR1
PD 173074 TTD 00010068	1/17	0.001699	0.019071	0	0	1248.875	7964.758	FGFR1
WZ-7043 LINCS	1/20	0.001999	0.019071	0	0	1051.526	6535.343	FGFR1
Kinome 511 Roche	1/20	0.001999	0.019071	0	0	1051.526	6535.343	FGFR1
CHEMBL1967116 Roche	1/21	0.002099	0.019071	0	0	998.9	6159.553	FGFR1
Lenvatinib FDA	1/21	0.002099	0.019071	0	0	998.9	6159.553	FGFR1
BMS-540215 Kinome Scan	1/22	0.002199	0.019071	0	0	951.2857	5821.717	FGFR1
SU-6668 MRC	1/23	0.002299	0.019071	0	0	908	5516.476	FGFR1
MAGNESIUM CTD 00006230	1/25	0.002498	0.019071	0	0	832.25	4986.91	SHC1
BX-912 MRC	1/26	0.002598	0.019071	0	0	798.92	4755.88	FGFR1
Pazopanib hydrochloride	1/28	0.002798	0.019071	0	0	739.6667	4348.373	FGFR1
NG-25 MRC	1/29	0.002898	0.019071	0	0	713.2143	4167.854	FGFR1
Compound C (Dorsomorphin) MRC	1/29	0.002898	0.019071	0	0	713.2143	4167.854	FGFR1
Kinome 635 Roche	1/29	0.002898	0.019071	0	0	713.2143	4167.854	FGFR1
BI-D-1870 MRC	1/29	0.002898	0.019071	0	0	713.2143	4167.854	FGFR1
PP242 MRC	1/30	0.002998	0.019071	0	0	688.5862	4000.606	FGFR1
TAE684 MRC	1/31	0.003098	0.019071	0	0	665.6	3845.251	FGFR1
IPA-3 MRC	1/33	0.003297	0.019071	0	0	623.9375	3565.584	FGFR1
Regorafenib FDA	1/33	0.003297	0.019071	0	0	623.9375	3565.584	FGFR1
SU 5416 TTD 00011104	1/34	0.003397	0.019071	0	0	605	3439.317	FGFR1
Quercetagetin MRC	1/36	0.003597	0.019071	0	0	570.3714	3209.886	FGFR1
SYK Inhibitor I MRC	1/36	0.003597	0.019071	0	0	570.3714	3209.886	FGFR1
R-406 MRC	1/36	0.003597	0.019071	0	0	570.3714	3209.886	FGFR1

Kinome 1194 Roche	1/37	0.003697	0.019071	0	0	554.5	3105.387	FGFR1
Axitinib FDA	1/39	0.003896	0.019071	0	0	525.2632	2914.026	FGFR1
AG-013736 Kinome Scan	1/39	0.003896	0.019071	0	0	525.2632	2914.026	FGFR1
MLN8054 LINCS	1/41	0.004096	0.019071	0	0	498.95	2743.119	FGFR1
TETRACHLOROETHYLENE CTD 00006849	1/41	0.004096	0.019071	0	0	498.95	2743.119	SHC1
BX-517 Analog (compound 7b) MRC	1/42	0.004196	0.019071	0	0	486.7561	2664.362	FGFR1
BIO (6-Bromoindirubin-3'-oxime) MRC	1/42	0.004196	0.019071	0	0	486.7561	2664.362	FGFR1
ACRYLAMIDE CTD 00007343	1/42	0.004196	0.019071	0	0	486.7561	2664.362	FGFR1
AZD-2171 Kinome Scan	1/46	0.004595	0.019841	0	0	443.4	2386.751	FGFR1
Crizotinib CTD 00004714	1/47	0.004695	0.019841	0	0	433.7391	2325.43	FGFR1
Pazopanib Kinome Scan	1/49	0.004894	0.019841	0	0	415.625	2211.015	FGFR1
Pazopanib FDA	1/49	0.004894	0.019841	0	0	415.625	2211.015	FGFR1
RO-31-8220 MRC	1/54	0.005393	0.019856	0	0	376.3208	1965.409	FGFR1
Vandetanib Kinome Scan	1/55	0.005493	0.019856	0	0	369.3333	1922.148	FGFR1
Vandetanib FDA	1/55	0.005493	0.019856	0	0	369.3333	1922.148	FGFR1
Sorafenib LINCS	1/56	0.005592	0.019856	0	0	362.6	1880.581	FGFR1
taurine CTD 00006829	1/57	0.005692	0.019856	0	0	356.1071	1840.612	FGFR1
BX-795 MRC	1/57	0.005692	0.019856	0	0	356.1071	1840.612	FGFR1
PONATINIB CTD 00004976	1/59	0.005891	0.019861	0	0	343.7931	1765.125	FGFR1
Sorafenib FDA	1/60	0.005991	0.019861	0	0	337.9492	1729.45	FGFR1
Electrocorundum CTD 00005364	1/61	0.006091	0.019861	0	0	332.3	1695.056	SHC1
WZ4002 LINCS	1/63	0.00629	0.019968	0	0	321.5484	1629.855	FGFR1
enoxaparin CTD 00006081	1/64	0.00639	0.019968	0	0	316.4286	1598.928	FGFR1
PARAOXON CTD 00006470	1/72	0.007187	0.022001	0	0	280.662	1385.197	FGFR1
hesperadin TTD 00008420	1/80	0.007984	0.023008	0	0	252.1392	1217.909	FGFR1
Decitabine CTD 00000750	2/1800	0.008096	0.023008	0	0	36400	175317.3	SHC1; FGFR1
KIN001-220 LINCS	1/82	0.008183	0.023008	0	0	245.8889	1181.658	FGFR1
Staurosporine MRC	1/83	0.008283	0.023008	0	0	242.878	1164.251	FGFR1
DL-Selenomethionine CTD 00006732	1/83	0.008283	0.023008	0	0	242.878	1164.251	SHC1
WH-4-025 LINCS	1/86	0.008582	0.023253	0	0	234.2706	1114.69	FGFR1
VX-680/MK-0457 Kinome Scan	1/87	0.008681	0.023253	0	0	231.5349	1099.002	FGFR1
Ponatinib FDA	1/90	0.00898	0.023336	0	0	223.6966	1054.231	FGFR1
sulpiride PC3 UP	1/91	0.009079	0.023336	0	0	221.2	1040.026	FGFR1
PKR Inhibitor MRC	1/92	0.009179	0.023336	0	0	218.7582	1026.16	FGFR1
Cdk1/2 Inhibitor III RBC	1/95	0.009478	0.023694	0	0	211.7447	986.4818	FGFR1

K252a MRC	1/101	0.010075	0.024774	0	0	198.98	914.857	FGFR1
CHIR-258/TKI-258 Kinome Scan	1/103	0.010274	0.024856	0	0	195.0588	893.0134	FGFR1
Axitinib	1/105	0.010473	0.024935	0	0	191.2885	872.0829	FGFR1
OTSSP167 MRC	1/108	0.010771	0.025245	0	0	185.8972	842.2812	FGFR1
chlorpromazine CTD 00005648	1/113	0.011268	0.025577	0	0	177.5536	796.4638	FGFR1
Sorafenib	1/116	0.011567	0.025577	0	0	172.8957	771.0522	FGFR1
PD-173955 Kinome Scan	1/116	0.011567	0.025577	0	0	172.8957	771.0522	FGFR1
Vandetanib	1/117	0.011666	0.025577	0	0	171.3966	762.8998	FGFR1
HG-5-113-01 LINCS	1/118	0.011765	0.025577	0	0	169.9231	754.8994	FGFR1
PP-242 Kinome Scan	1/121	0.012064	0.025851	0	0	165.65	731.7695	FGFR1
N-NITROSODIMETHYLAMINE CTD 00005845	1/126	0.012561	0.026218	0	0	158.984	695.9044	SHC1
solanine HL60 UP	1/127	0.01266	0.026218	0	0	157.7143	689.1038	FGFR1
AZD4547 LINCS	1/128	0.012759	0.026218	0	0	156.4646	682.4201	FGFR1
TG-101348 Kinome Scan	1/134	0.013355	0.026909	0	0	149.3609	644.6178	FGFR1
WH-4-023 LINCS	1/135	0.013455	0.026909	0	0	148.2388	638.6766	FGFR1
BMS-536924,KIN001-126 LINCS	1/142	0.01415	0.02749	0	0	140.8298	599.6608	FGFR1
HG-6-64-01 LINCS	1/145	0.014448	0.02749	0	0	137.875	584.207	FGFR1
Dasatinib	1/145	0.014448	0.02749	0	0	137.875	584.207	FGFR1
ellagic acid CTD 00005891	1/146	0.014547	0.02749	0	0	136.9172	579.2112	FGFR1
AST-487 Kinome Scan	1/148	0.014745	0.02749	0	0	135.0408	569.4426	FGFR1
EXEL-2880/GSK-1363089 Kinome Scan	1/149	0.014845	0.02749	0	0	134.1216	564.6667	FGFR1
HG-9-91-01 LINCS	1/153	0.015242	0.027773	0	0	130.5658	546.2505	FGFR1
dmnq CTD 00002569	1/157	0.015639	0.027773	0	0	127.1923	528.867	SHC1
BIBF-1120 (derivative) Kinome Scan	1/158	0.015738	0.027773	0	0	126.3758	524.6727	FGFR1
Nintedanib FDA	1/158	0.015738	0.027773	0	0	126.3758	524.6727	FGFR1
Adehl CTD 00006002	1/163	0.016234	0.028315	0	0	122.4444	504.5515	FGFR1
Coxistac CTD 00000539	1/169	0.016829	0.029015	0	0	118.0357	482.1356	FGFR1
hydrogen peroxide CTD 00006118	2/2673	0.017856	0.030347	0	0	34654	139496	SHC1; FGFR1
KW-2449 Kinome Scan	1/184	0.018316	0.030347	0	0	108.2787	433.1146	FGFR1
Copper(II) chloride CTD 00001408	1/184	0.018316	0.030347	0	0	108.2787	433.1146	FGFR1
Sunitinib Kinome Scan	1/185	0.018415	0.030347	0	0	107.6848	430.158	FGFR1
Sunitinib FDA	1/187	0.018613	0.030347	0	0	106.5161	424.3497	FGFR1
R406 LINCS	1/194	0.019306	0.031139	0	0	102.6166	405.0612	FGFR1
Staurosporine, Streptomyces sp. RBC	1/197	0.019603	0.031282	0	0	101.0306	397.2581	FGFR1

R406 Kinome Scan	1/203	0.020197	0.031352	0	0	98	382.4161	FGFR1
HG-14-10-04 LINCS	1/204	0.020296	0.031352	0	0	97.51232	380.0363	FGFR1
TAE-684 Kinome Scan	1/204	0.020296	0.031352	0	0	97.51232	380.0363	FGFR1
BX-912 LINCS	1/206	0.020494	0.031352	0	0	96.55122	375.3535	FGFR1
Sodium dichromate CTD 00000827	1/208	0.020692	0.031352	0	0	95.6087	370.7704	FGFR1
NVP-TAE684 LINCS	1/212	0.021088	0.031632	0	0	93.77725	361.8912	FGFR1
Aflodac CTD 00006817	1/234	0.023264	0.034455	0	0	84.82833	319.0282	SHC1
OTSSP167 LINCS	1/237	0.02356	0.034455	0	0	83.73729	313.8645	FGFR1
midecamycin HL60 DOWN	1/238	0.023659	0.034455	0	0	83.37975	312.1754	SHC1
AZD7762 LINCS	1/254	0.025239	0.036403	0	0	78.04348	287.1499	FGFR1
Sunitinib	1/258	0.025634	0.03662	0	0	76.81323	281.4309	FGFR1
CEP-701 Kinome Scan	1/272	0.027016	0.038149	0	0	72.79336	262.8818	FGFR1
2-Nonenal, 4-hydroxy-, (2E,4R)- CTD 00001295	1/274	0.027213	0.038149	0	0	72.25275	260.4038	FGFR1
chlortetracycline HL60 UP	1/289	0.028692	0.03985	0	0	68.4375	243.0316	FGFR1
OCHRATOXIN A CTD 00001202	1/297	0.02948	0.040569	0	0	66.56081	234.5631	SHC1
Staurosporine Kinome Scan	1/315	0.031253	0.042617	0	0	62.6879	217.2546	FGFR1
MERCURY CTD 00006274	1/364	0.036069	0.048743	0	0	54.09091	179.7067	SHC1
ampyrone HL60 UP	1/380	0.03764	0.05041	0	0	51.76517	169.774	FGFR1
thapsigargin MCF7 UP	1/407	0.040287	0.053478	0	0	48.25616	154.986	SHC1
diclofenac CTD 00005804	1/427	0.042245	0.055586	0	0	45.94366	145.3781	FGFR1
acetaminophen CTD 00005295	2/4135	0.042737	0.055744	0	0	31730	100034.7	SHC1; FGFR1
carbamazepine CTD 00005574	1/436	0.043126	0.055766	0	0	44.97241	141.377	FGFR1
estradiol CTD 00005920	2/4336	0.046994	0.060248	0	0	31328	95793.04	SHC1; FGFR1
Phorbol 12-myristate 13-acetate CTD 00006852	1/483	0.047718	0.060658	0	0	40.48963	123.1878	SHC1
benzo[a]pyrene CTD 00005488	2/4424	0.048921	0.061665	0	0	31152	94002.92	SHC1; FGFR1
Tamibarotene CTD 00002527	1/559	0.05512	0.0689	0	0	34.83871	100.9711	FGFR1
etifenin PC3 DOWN	1/609	0.059974	0.074348	0	0	31.89145	89.73755	FGFR1
methotrexate CTD 00006299	1/619	0.060943	0.074676	0	0	31.35922	87.73717	FGFR1
ambroxol PC3 DOWN	1/622	0.061234	0.074676	0	0	31.2029	87.15132	SHC1
scriptaid PC3 UP	1/664	0.065299	0.078991	0	0	29.1629	79.57904	FGFR1
anisomycin MCF7 UP	1/718	0.070513	0.084615	0	0	26.89121	71.3145	SHC1
Vitinoin CTD 00007069	1/780	0.076481	0.090445	0	0	24.67137	63.42315	FGFR1
ampyrone HL60 DOWN	1/781	0.076577	0.090445	0	0	24.63846	63.3076	SHC1
tamoxifen CTD 00006827	1/802	0.078594	0.092058	0	0	23.96629	60.95741	FGFR1
Caspan CTD 00000180	1/808	0.07917	0.092058	0	0	23.78067	60.31168	SHC1
captopril PC3 DOWN	1/856	0.08377	0.096658	0	0	22.38947	55.51876	FGFR1

Bortezomib CTD 00003736	1/878	0.085875	0.09833	0	0	21.80274	53.52282	SHC1
phenobarbital CTD 00006510	1/1129	0.109716	0.124677	0	0	16.72872	36.96818	SHC1
CADMIUM CTD 00005555	1/1142	0.110942	0.125123	0	0	16.52673	36.33812	SHC1
genistein CTD 00007324	1/1231	0.119314	0.133561	0	0	15.25854	32.43957	SHC1
Bisphenol A CTD 00000312	1/1262	0.122221	0.135801	0	0	14.85884	31.23216	FGFR1
Arsenenous acid CTD 00000922	1/1283	0.124187	0.136971	0	0	14.59906	30.45311	FGFR1
arsenite CTD 00000779	1/1300	0.125778	0.137713	0	0	14.39492	29.84411	SHC1
Disodium selenite CTD 00007229	1/1370	0.132311	0.143816	0	0	13.60774	27.52306	SHC1
SELENIUM CTD 00006731	1/1559	0.149827	0.161684	0	0	11.83569	22.46737	FGFR1
resveratrol CTD 00002483	1/1601	0.153695	0.164674	0	0	11.49875	21.53466	FGFR1
COUMESTROL CTD 00005717	1/1812	0.172995	0.184038	0	0	10.04252	17.6195	FGFR1
AFLATOXIN B1 CTD 00007128	1/3081	0.284375	0.299448	0	0	5.492857	6.907062	SHC1
7646-79-9 CTD 00000928	1/3094	0.285474	0.299448	0	0	5.465567	6.851658	SHC1
formaldehyde CTD 00006001	1/3192	0.293734	0.305973	0	0	5.267001	6.452498	FGFR1
0175029-0000 PC3 DOWN	1/3326	0.304951	0.315466	0	0	5.014436	5.955168	FGFR1
Tetradioxin CTD 00006848	1/3768	0.341313	0.350664	0	0	4.308734	4.6317	FGFR1
Retinoic acid CTD 00006918	1/4258	0.380482	0.388247	0	0	3.697674	3.573128	FGFR1
cyclosporin A CTD 00007121	1/4825	0.424307	0.430041	0	0	3.145522	2.696649	SHC1
Copper sulfate CTD 00007279	1/6016	0.511129	0.51456	0	0	2.324688	1.560173	SHC1
VALPROIC ACID CTD 00006977	1/8312	0.658488	0.658488	0	0	1.406209	0.587526	FGFR1
